# The Current Challenges for Drug Discovery in CNS Remyelination

**DOI:** 10.3390/ijms22062891

**Published:** 2021-03-12

**Authors:** Sonia Balestri, Alice Del Giovane, Carola Sposato, Marta Ferrarelli, Antonella Ragnini-Wilson

**Affiliations:** Department of Biology, University of Rome “Tor Vergata”, Viale della Ricerca Scientifica, 00133 Rome, Italy; sonia.balestri@alumni.uniroma2.eu (S.B.); alice.delgiovane@alumni.uniroma2.eu (A.D.G.); carola.sposato@alumni.uniroma2.eu (C.S.); marta.ferrarelli@alumni.uniroma2.eu (M.F.)

**Keywords:** oligodendrocytes, microfibers, remyelination, 3d scaffolds, drug screen

## Abstract

The myelin sheath wraps around axons, allowing saltatory currents to be transmitted along neurons. Several genetic, viral, or environmental factors can damage the central nervous system (CNS) myelin sheath during life. Unless the myelin sheath is repaired, these insults will lead to neurodegeneration. Remyelination occurs spontaneously upon myelin injury in healthy individuals but can fail in several demyelination pathologies or as a consequence of aging. Thus, pharmacological intervention that promotes CNS remyelination could have a major impact on patient’s lives by delaying or even preventing neurodegeneration. Drugs promoting CNS remyelination in animal models have been identified recently, mostly as a result of repurposing phenotypical screening campaigns that used novel oligodendrocyte cellular models. Although none of these have as yet arrived in the clinic, promising candidates are on the way. Many questions remain. Among the most relevant is the question if there is a time window when remyelination drugs should be administrated and why adult remyelination fails in many neurodegenerative pathologies. Moreover, a significant challenge in the field is how to reconstitute the oligodendrocyte/axon interaction environment representative of healthy as well as disease microenvironments in drug screening campaigns, so that drugs can be screened in the most appropriate disease-relevant conditions. Here we will provide an overview of how the field of in vitro models developed over recent years and recent biological findings about how oligodendrocytes mature after reactivation of their staminal niche. These data have posed novel questions and opened new views about how the adult brain is repaired after myelin injury and we will discuss how these new findings might change future drug screening campaigns for CNS regenerative drugs.

## 1. Introduction

Myelin regenerative medicine aims to improve the spontaneous remyelination potential of the adult CNS. Axon–glial interactions represent one of the most complex cell-to-cell communication systems in the human body, thereby presenting several challenges for drug discovery. How neurons and the myelinating cells of the CNS, the oligodendrocytes (OLs), crosstalk and how this interaction evolves during acute and chronic pathological phases of demyelination diseases are matters of several ongoing studies [1,2,3,4,5]. Recent studies have provided a wealth of information providing the prospect of halting and eventually reversing the effects of devastating demyelination pathologies using pharmacological approaches [6,7,8,9].

CNS human pathologies that cause primary or secondary demyelination can be of genetic, viral, or environmental origin. Among the acquired non-traumatic demyelination pathologies is multiple sclerosis (MS). MS is an inflammatory demyelinating disease that leads to neurodegeneration as a consequence of an autoimmune attack on the CNS myelin. MS is one of the most frequent neurological diseases in young adults (50–100 cases in 100,000 individuals) in Western countries [10]. Current immunomodulatory therapies can reduce or halt disease progression, but they cannot prevent the accumulation of permanent disability consequent to demyelination of axons [10,11]. Other CNS pathologies classically considered strictly neurodegenerative that, however, present nerve demyelination at early stages of disease are amyotrophic lateral sclerosis (ALS) [12], schizophrenia (SCZ), [13,14,15,16], Alzheimer’s disease (AD), Parkinson’s disease (PD), and pathologies linked to the spectrum of autistic dysfunctions (ASD) [17]. Last but not least, brain aging and age-related dementia could potentially be delayed using remyelination agents [18]. 

The increasing molecular understanding of how demyelination and remyelination occur in healthy individuals (e.g., during aging) as well as in various pathological conditions has increased the interest in developing pharmacological approaches to adult CNS remyelination. Clearly, to be effective at the clinical level, remyelination compounds should be evaluated in the context of disease progression [1,2,18,19]. However, the complexity of studying brain tissues in vivo using imaging [20,21], combined with the fact that murine brain development does not completely reflect human brain aging or demyelinating pathology progression [22,23], poses the problem of how to validate molecular data obtained in animal model. Last but not least, the question of what the potential window for remyelination treatment is remains unanswered. How to follow clinical progression during clinical trials is a major issue in CNS remyelination treatments due to the lack of accessible tissues for histological examination and of myelin regeneration biomarkers in biological fluids, so further development of in vivo image-based assessments of nerve remyelination is paramount. A recent review discusses the new development of neuroimaging for MS treatments, and therefore this aspect will not be discussed here [24]. Here we want to provide a general picture of the many factors contributing to remyelination and their relevance for drug discovery. Many biophysical and physiological aspects of adult CNS remyelination in humans remain unclear, even in healthy individuals: multiple cell types (neurons, astrocytes, microglia) are clearly involved in the orchestration of the events leading to axon remyelination. The cooperative action of multiple cell types at lesions poses the problem of identifying which of them (if any) are primarily affected in the specific demyelination pathology under study. Most of the literature so far is based on rodent animal models [11]. New data based on single cell analyses have revolutionized our understanding about how oligodendrocyte precursor cells (OPC) differentiate to mature OLs in man compared to mice [3,5,25]. 

The complexity of in vivo remyelination studies when testing drugs makes in vivo approaches unfeasible and several reductionist approaches have been undertaken to reconstitute the three-dimensional (3D) environment in which remyelination occurs. The aim is to refine the activity on targets in cell lines or organoids and clarify drug molecular action in each step of OPC differentiation prior to in vivo studies to reduce development costs and drug failure at later stages. Remyelination agents so far identified can overcome one or multiple stages of OPC development impairment. We hope that biomarkers that stratify patients according to their demyelination defects will be developed soon and applied at the clinical level. 

Various in vitro technologies have been developed to recreate different 3D aspects of the axon/glia interaction. Here, we will review the most successful that have been used for drug screening or drug validation. Artificial axons have been made of different materials and can be electrospun or printed in regular arrays in growth chambers and multi-well plates. The artificial axon-containing plates support oligodendrocyte (OL) growth and oligodendrocyte precursor cell (OPC) differentiation even better than 2D cultures [26,27,28]. Multi-well platforms with micropillars, to mimic neurons, permitted several drug library screens to be performed to select drugs that more specifically promote axon engagement by OLs [29,30]. Collectively, studies on the mechanisms of how mature OLs engage axons in the absence of neuronal feedback combined with the development of imaging software supported by artificial intelligence have provided new views on how OLs myelinate axon fibers in vivo [1,2,21,22,23,25,26,27,28]. Novel 3D biomaterials suitable for studying oligodendrocyte and axon interactions are under development to improve the mechano-chemical properties of artificial axon fibers and cone supports and make them more similar to axons during disease development [27]. Bioprinting of neuronal tissues and organoids are the newest frontier in developments for drug screening and validation studies of remyelination agents and will open new avenues in the field [31,32]. Altogether, these studies have shown that it is possible to pharmacologically stimulate remyelination in animal models by screening drug libraries in in vitro platforms [6,7,29,30,33,34]. 

The wealth of data obtained in in vitro models has helped in our understanding of the details of the remyelination process in vivo. New single cell analyses from the brains of rodent models and postmortem human samples and the development of isolation technologies allowing to derive OPCs from patient iPSCs will further improve future drug discovery programs [3,4,23]. The detailed description of the compound classes selected by the different screens and their prospective use in the clinic have been covered in many recent reviews [10,11,35,36]. 

Here, we review the main contributions that have led to the current state-of-the-art of in vitro models, drug screening, and validation studies. Although at the moment it is early to evaluate how these findings will impact clinical therapies (Table 1 and Table 2), they have provided a wealth of knowledge on the mechanisms of adult CNS remyelination [1,4,6,7,9,37]. Furthermore, we discuss recent biological findings in the CNS remyelination process, as we think new drug discovery programs will have to focus on recent advances in the molecular understanding of OPC differentiation in man compared to murine models. We think that these new biological findings combined with the use of 3D models will change future drug screening approaches, positively impacting on the quality of selected drugs for human CNS remyelination. 

## 2. The Biological Basis of CNS Remyelination 

In humans, myelination starts in the embryo and terminates during adolescence. In adults, myelin remodeling and adaptive myelination have been correlated with activity-dependent changes at synapses and have been demonstrated in mice during motor skill learning [62,63,64]. Supporting the view that remyelination of axons in the adult brain is a fundamental process for brain health, white matter loss correlates with cognitive decline during aging and Alzheimer’s disease progression [65,66]. Remyelination restores nerve conductance after a demyelination event and prevents neurodegeneration, restoring nerve functionality. There is wide variability in the remyelination response among MS patients, but in all cases, this declines with age [10,11]. Other neurodegeneration pathologies showing demyelination at early stage are ALS, PD, SCZ, and some pathologies with the spectrum of ASD. These are likely to provide new models in which to test remyelination agents for their ability to delay neurodegeneration by promoting remyelination [12,13,16,17].

The main difficulty in studying the demyelination and remyelination processes comes from the multiple cell types that participate in these processes, the difficulty in accessibility of tissue and white matter samples, and, last but not least, the lack of animal disease models reflecting human disease progression. Physiopathological studies in autopsy samples from MS patients have shown that demyelination of the upper layers of the cortex correlates with regions of leptomeningeal inflammation, composed of B and T cells that secrete cytotoxic cytokines and create a complex inflammatory milieu that contains macrophages and activated microglia at the active demyelinating regions [67,68]. Several studies have shown that within the context of an acute inflammatory response, such as that present at the initial stages of MS, there are myelin-laden macrophages at the lesion edge and various chemokines produced by astrocytes. 

Astrocytes have important roles in synaptogenesis, neurotransmission, and blood–brain barrier (BBB) formation and maintenance. In response to neuroinflammation, astrogliosis is observed. Reactive astrocytes can be either beneficial or detrimental during remyelination, since they can induce oligodendrocyte precursor cell (OPC) maturation but also oligodendrocyte and neuronal apoptosis [69,70].

**Figure 1:**
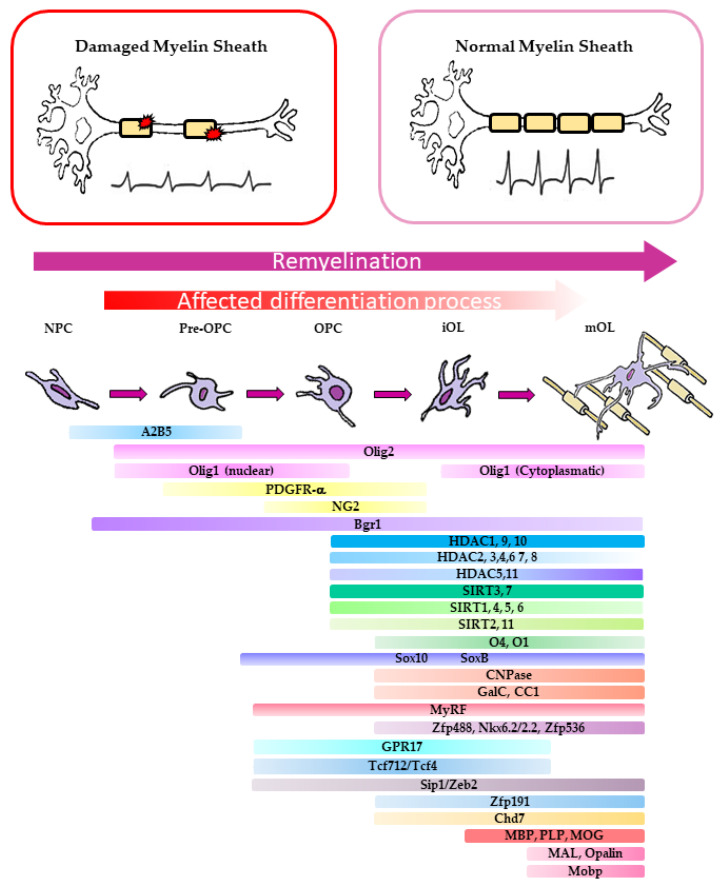
Remyelination is due to new oligodendrogenesis and differentiation of OPCs to mature and myelinating OLs. The remyelination process is activated in the adult brain during the processes of myelin turnover, myelin plasticity, or in the event of damage. This process involves oligodendrogenesis, the activation of neural progenitor cells (NPCs), proliferation and differentiation to mature OLs (mOL), or reactivation of resident oligodendrocyte precursor cells (OPCs), quiescent from the phase of embryonic brain development, which can differentiate into mature and myelinating OLs. This complex process of differentiation, driven by as yet not fully understood mechano-biological signaling, decreases with demyelinating disease progression and during aging. There is a strong effort to isolate drugs able to restore the remyelination process, starting from both the OPC and NPC remyelinating pools. The numerous studies carried out have characterized the main factors, listed in the figure, that distinguish each development phase of the OL lineage. iOL—immature oligodendrocytes; pre-OPCs—pre-oligodendrocyte precursor cells. Color-bars intensity indicates level of expression

Cell-fated neural stem cells (NSCs) committed to oligodendrogenesis are reactivated under remyelination stimuli. They differentiate in OPCs that repopulate the demyelinated area, where they mature in myelinating oligodendrocytes (OL) (Figure 1). A heterogeneous population of OPCs repopulate the demyelinated area in healthy individuals, but the OPC pool is depleted over time at chronic MS lesions and in aged patients. The reason(s) for the decrease in OPC differentiation in mature OLs has been the matter of several studies and the factors contributing or impairing OPC differentiation represent the main targets of drugs selected for remyelination therapy (Table 1 and Table 2) [5,9,19]. 

The contribution of the inflammatory environment to OPC differentiation defects has been indicated by many studies as a major factor leading to remyelination defects in MS patients [4,5,9,71]. The presence of debris-filled macrophages promotes remyelination in toxin-induced demyelination models. Their beneficial role at lesions has been attributed to the ability of macrophages to clear up the myelin debris generated during demyelination, by phagocytosis, as well as to their ability to secrete various factors influencing OPC maturation (CXCR4, tumor necrosis factor, endothelin, and activin-A). Macrophages may also remodel the metabolic support of axons and oligodendrocytes, and the time of transition between the M1 and M2 state can change the mature OLs at lesions. The M1 state is prevalent during the OPC recruitment phase of remyelination, whereas the M2 state is the main state observed during the OPC differentiation phase. Additionally, the age-related decline of remyelination appears to depend in part on a decline in the efficiency of the innate immune system to switch from a predominantly M1 population to a predominantly M2 population [70,71,72]. Using heterochronic parabiosis, in which two adult mice of different ages are joined (such that they share a common circulation), it has been shown that deficient remyelination in an aged mouse can be reversed. These data validated the idea that targeting endogenous OPC differentiation, even in aged patients, might reverse the acquired remyelination defects [73]. 

Pharmacological intervention has since then focused on the identification and pharmacological targeting of the main factors involved in the regulation of OPC recruitment and differentiation (Figure 1). Below, we will discuss the potential drug targets identified by biological studies affecting OPC recruitment and differentiation.

### 2.1. Molecular Aspects of OPC Differentiation in Murine Models of Demyelination

Due to an intrinsic limited availability of primary OLs from MS patients, most available studies on the molecular aspects of OPC differentiation into mature OLs have been performed in murine models of demyelination: e.g., cuprizone-fed mice, experimental autoimmune encephalomyelitis (EAE), or focal demyelination induced by stereotaxic lysophosphatidylcholine (LPC) injection [11,74]. 

Regions of myelin remyelination can be distinguished from old myelinated neurons as they differ in thickness [75]. To clarify the dynamics of OLs in the mature brain of mice, two photon fluorescence microscopy high-resolution imaging was used. This study defined the extent of myelin remodeling during myelin regeneration and the specificity of myelin replacement within the adult somatosensory cortex after demyelination in cuprizone-fed transgenic mice expressing EGFP-myelin-associated oligodendrocyte basic protein (Mobp-EGFP) [76]. The authors showed that cuprizone-treated mice never reach a normal oligodendrocyte density in the deeper layer of the cortex after demyelination. Interestingly, new myelin is regenerated but in regions distinct from those occupied before injury. Changes in oligodendrogenesis can alter the behavioral performance of mice. It remains unclear if the reformed myelin region fully restores lost function even if the precise pattern of myelination is changed [75]. 

In rodents, remyelination typically initiates with the mobilization of quiescent lineage-specific NSCs from the subventricular zone (SVZ) of the corpus callosum [77]. These NSCs are committed to the oligodendrocyte (OL) lineage. They differentiate into OPCs under the control of mitogenic growth factors, such as epidermal growth factor (EGF), fibroblast growth factor (FGF), and plated-derived growth factor (PDGF), and morphogenetic factors such as Sonic Hedgehog (Shh) [17,78,79,80]. EGF expression can influence cortical progenitor fate choice during the neural progenitor cell (NPC) proliferative stage [81,82]. FGF2 promotes specification and proliferation of mouse-derived cells but not of human-derived precursors [83]. During remyelination, morphogenic Hedgehog (Hh) signaling is reactivated in the SVZ staminal niche where NPC proliferation is observed [84,85,86,87]. Consistent with this, Sonic Hedgehog (Shh) administration increases OPCs and pre-OLs in the adult healthy dorsal forebrain [88]. Oligodendrogenesis in the SVZ is regulated by activation of the Shh morphogen by releasing the Patched receptor (PATCH) from Smoothened (Smo) receptor inhibition. This event initiates non-canonical Smo activation and signaling involving Glioma-associated oncogene protein 1 (Gli1) downregulation [79,89,90,91]. Smo is a G protein-coupled receptor (GPCR) consisting of seven transmembrane domains, belonging to the Frizzled family, that responds to an increase in Shh. In the canonical Smo activation pathway, the interaction of the Shh ligand with PTCH results in Smo migration to the cilium, a process that leads to a chain of events that culminates in a change in the balance of activating and repressor forms of Gli factors (GLI1, 2, and 3) [78,92]. GLI proteins can activate or repress gene transcription, regulating cell differentiation and proliferation [92]. Gli1 upregulation is typically observed upon activation of Shh/Smo signaling. However, during NPC commitment to OPCs, Gli1 is repressed upon Shh/Smo activation [90]. Why and how, during remyelination, Shh/PITCH/Smo signaling results in Gli1 downregulation remains to be clarified [78,79,90]. The role of Smo activation during OPC differentiation until OL maturation remains to be determined. The idea that Smo activation is required also during OPC differentiation to mature OLs is suggested by the identification of several anti-inflammatory drugs with Smo agonist activity in phenotypical screens for remyelination agents [7,33,78] (Table 2).

Further maturation and differentiation of OPCs involves multiple transitions through progenitor pro-myelinating (pre-OL), myelinating (OL), and mature OLs (Figure 1). Each of these OPC differentiation stages can be distinguished by the expression of specific sets of transcription factors (TF) required for myelin protein expression, inhibition of apoptosis, and for the morphological changes of the plasma membrane occurring during axon engagement [93,94] (Figure 1).

In cuprizone mice, following myelin injury, the rapid mobilization of OPCs from the corpus callosum to the demyelination area promotes a temporally and spatially orchestrated activation of a complex panel of lineage-specific TFs, including Olig1-2, MyrF, Sox 17 and Sox10, the inhibition of adhesion G protein-coupled receptors, and Wnt signaling [95]. MyRF-regulated gene transcription, together with chromatin remodeling factors, regulates the step-wise oligodendrocyte lineage differentiation process of primitive oligodendrocyte progenitor cells (pre-OPCs), also known as uncommitted OPCs (Olig1/2+/A2B5+/PDGFRαlow), to the committed OPCs expressing high levels of PDGFRα and NG2. OPCs differentiate into immature oligodendrocytes (pre-OLs) that express O4+ and the myelin protein CNP. Finally, they differentiate in oligodendrocytes (CC1+) and finally into mature myelinating oligodendrocytes, expressing myelin proteins (e.g., MBP+/PLP+/MOG+) [93]. The myelin regulatory factor MyRF has been shown to be a master regulator of OPC to pre-OL differentiation and motor learning capability in adult mice [62,63], being required for the expression of the vast majority of CNS myelin genes but not for OPC specification or differentiation per se [96]. Retinoid X Receptor gamma (RxRγ) is among the transcripts differentially expressed in the remyelination area promoting OPC recruitment and remyelination. Mice that lack RxRγ fail in adult oligodendrogenesis [38]. Consistent with this finding, many of the promyelinating drugs isolated in phenotypical screens upregulate both MBP expression and RxRγ [33,37]. Using purified primary OPCs isolated from control and OPC-specific Chd7-deficient mice, it has been shown that TFs such as Sox10, Olig2, Nkx2.2, and Ascl1 are key regulators of OL differentiation by directly controlling transcription of genes implicated in chromatin remodeling. A growing body of evidence suggests that some of these TFs work together with SWI/SNF chromatin remodeling and cooperate to promote the expression of OPC differentiation genes. These findings open the possibility that resident OLs might undergo an epigenetic change orchestrated by TFs known to regulate OL differentiation [94]. 

RiboTag technology has been used to determine OL lineage cell-specific gene expression in vivo in the corpus callosum of chronic cuprizone and EAE mouse models. This study showed that up-regulation of cholesterol synthesis pathways dominated the transcriptome profile of oligodendrocyte lineage cells during remyelination after injury. Consistent with this, cholesterol synthesis gene expression is decreased at the whole tissue level in human autopsy tissues of MS patients compared with normal controls and in the optic chiasm, spinal-cord astrocytes, and oligodendrocytes in EAE mice compared with normal mice [97]. It has been observed that increased expression of the ATP-binding cassette transporter A1 (ABCA1) in astrocytes correlates with increased cholesterol synthesis and improved EAE clinical scores compared to controls, although MBP staining did not increase in oligodendrocytes. Similarly, estrogen receptor β-ligand treatment up-regulated cholesterol synthesis pathways in oligodendrocytes and increased remyelination in EAE mice [97]. Together these data support the view that up-regulation of cholesterol synthesis pathways in oligodendrocyte lineages during remyelination is beneficial for regeneration after demyelination. While this might suggest that dietary cholesterol could enhance remyelination, it is known that it can lead to adverse cardiovascular effects and comorbidities in patients with MS. On the other hand, cardiovascular treatments such as statins, common in elderly people, might be deleterious for remyelination, so they should be considered as a potential confounding variable in patients enrolled in future clinical trials of neuro-protective treatments for MS. Drugs that promote remyelination by regulating cholesterol biosynthetic pathway intermediates have been identified in drug library phenotypical screens. However, their efficacy in the clinic remains to be determined [98] (Table 1 and Table 2). 

Myelin proteins (MBP, PLP, CNPase, and MOG) are synthetized after MyRF/Sox10 expression, marking the passage of pre-OLs to myelinating OLs. The expression of the cytosolic MBP unlocks the cofilin-mediated membrane enlargement required for axon engagement [99,100]. Studies of mutations in the CNPase and PLP1 genes indicated their role in regulating axonal trafficking at the node of Ranvier. The first stage of axon engagement is independent of neuronal feedback but depends on the diameter of the axon to be wrapped [26,82]. Phosphatidylinositol 4,5-bisphosphate (PIP2) is the substrate of phosphoinositide 3-kinase (PI3K) that uses it to produce phosphatidylinositol (3,4,5)-trisphosphate (PIP3). PI3K/PIP3/AKT is the main signaling pathway activated by EGFR-TK upon ligand binding in mature OLs. Inhibitors of EGFR1 (erlotinib and gefitinib) have been selected in phenotypical screens and been shown to promote OPC differentiation in vitro by inhibiting PI3K/AKT signaling and by promoting PIP2 upregulation and binding to MBP at the peak of MBP expression, an event that signs the initiation of membrane enlargement and axon engagement [37,100]. How axon ensheathment proceeds after MBP/PIP2 binding remains to be fully understood at the molecular level [82,100]. 

The positive regulators of OPC differentiation compete with signals that inhibit OPC differentiation such as canonical β-catenin–Wnt and Notch1–Jagged1 signaling pathways [93]. LINGO-1 (leucine rich repeat and immunoglobin-like domain-containing protein 1) binds to Erb2 and promotes increased translocation of this tyrosine kinase receptor into lipid rafts [101]. BIIB033, an anti-LINGO1 monoclonal antibody, entered into phase II studies with the name opicinumab by Biogen [49,50,102] (Table 1), but the efficacy of this drug remains to be clarified [11]. LINGO 1, myelin debris, inflammatory cytokines, and Nogo factors are only some of the elements recently identified as key components of the machinery that fine-tunes myelination of axons [80,103,104,105]. How positive and negative factors are balanced in normal and pathological conditions remains unclear [19,105,106]. 

### 2.2. Molecular Basis of Remyelination Defects in MS Patients

Altogether, the studies performed in rodent models for MS have suggested that either NPC differentiation to OPCs or OPC differentiation defects might be the main reason for the lack of remyelination in human white matter of chronic MS patients [9,19] (Figure 1). Recently, this idea has been challenged by single cell analyses of post-mortem MS samples [107,108]. These studies showed that MS patients OPCs at lesions have a variegated transcriptional expression: this indicates a higher OPC heterogeneity with respect to what was previously thought. Specifically, RNA sequencing of single nuclei (snRNA-seq) from the white matter areas of the human brain of post-mortem control and MS patients identified OL sub-clusters in the white matter area of controls, but new markers for these cell states were not present in MS samples. Analysis of differential gene expression showed that several myelin protein mRNAs are upregulated in mature OLs of MS patients compared to controls. This may indicate that MS disease progression might lead to an increase in the transcription of genes involved in myelination, indicating the existence of populations of mature OLs with more heterogeneous characteristics compared to controls [107]. In other words, the OL heterogeneity observed at lesions in MS patients might arise from the requirement to change the pathways activated during OPC differentiation, as a consequence of remyelination failure. Moreover, it appears that resident OLs might also contribute to the remyelination process at lesions [23,108]. Indeed, these observations are consistent with a previous single cell mRNA sequencing study performed in mice. It was shown that OL mRNA isolated from 10 distinct regions of the anterior–posterior and dorsal–ventral axis of murine juvenile and adult CNS identified thirteen distinct OL populations, twelve of which represented a continuum from PDGFRα+ OPCs to distinct mature oligodendrocytes [109]. Differentiation-committed oligodendrocyte precursors (COPs) were distinct from OPCs because they lacked PDGFRα and Cspg4 markers and expressed Neu4 and genes involved in keeping OLs undifferentiated (Sox6, Bmp4, and Gpr17). Myelin-forming OLs were identified based on their ability to express genes responsible for myelin formation (Mal, Mog, Plp1, Opalin, and Serinc5). Unfortunately, although these data indicated that murine OLs are a morphologically heterogeneous population, at this stage of the study it was not possible to define which population contributed to the remyelination lineage. Thus, it remained unclear whether OLs become morphologically diversified during OPC maturation through interactions with the local environment or whether there is intrinsic functional heterogeneity in OLs in mice [109].

Another study performed using adult human brain OPC cells transplanted into the CNS of myelin deficient mice showed that resident OPCs change their global transcription profile moving toward a profile that more closely resembles a neonatal OPC profile [110]. Consistent with this study, it has been possible to identify and isolate a putative progenitor cell population that resembles cells referred to as pre-oligodendrocytes by using anti-A2B5 antibody recognizing cell surface-expressed gangliosides. The A2B5 antibody-selected cells (A+) isolated from the adult human brain and transplanted into the CNS of myelin deficient mice are capable of myelination [111]. This study showed also that A2B5+ cells have a greater ability to engage microfibers and enlarge their membranes laterally along the fibers when cultured for six days in the presence of artificial axons (see below). 

Yet another study used magnetic resonance imaging (MRI) to longitudinally track inflammatory demyelinating lesions in post-mortem human white matter tissues (PMWM) from MS patients. The dynamics of remyelination in the normal-appearing white matter (NAWM) were retrospectively analyzed by measuring the integration of 14C derived from nuclear testing in genomic DNA in post-mortem human brain tissue [112]. Surprisingly, few new oligodendrocytes in shadow plaques were observed, suggesting that remyelination of lesions occurs transiently or not at all, or that myelin is regenerated by pre-existing, and not new, oligodendrocytes in the human brain. If shadow plaques represent remyelinated areas in the human brain, these data would indicate that remyelination is carried out by old, spared OLs in MS patients. This implies that current animal models of demyelination and remyelination may not optimally reflect the development of MS in the human brain, or it should be accepted that remyelination can be accomplished in diverse ways, depending on the injury model [112]. However, it is possible that the low spatial resolution of standard MRI images [113] and the indirect nature of MRI methods [103] might not be optimal to assess the origin of OLs [114]. 

Bribián and colleagues [3] further investigated the differences between the mouse and human adult brain with respect to the respective OPC migratory properties. They showed that murine and human OPCs have similar migratory properties in response to FGF2 and anosmin-1 but differ from that of OPCs taken from early post-natal samples [3]. Altogether, these data are consistent with the observation that the markers expressed by oligodendrocyte populations at lesions change according to the inflammatory milieu and support the view that inflammation has important effects on the remyelination capability of OLs. Whether a cell-mediated immune response, the local release of cytotoxic compounds, or environmental changes substantially shift the burden of repair from OPCs to surviving oligodendrocytes in human MS remains to be explored [23,107,112,114]. 

## 3. Influence of the Three-Dimensional (3D) Environment on the Axon Myelination 

Oligodendrocyte processes are highly motile extensions sensing the neighboring cells to ensure uniform spacing of myelin segments with evenly spaced nodes [115,116]. A number of different models have been proposed to explain how a myelin sheath might engage axons during development. The “carpet crawler” model [117,118] proposes that the oligodendrocyte membrane broadens and extends along the entire axonal segment prior to turning and moving underneath the growing sheet. However, it is to be expected that the molecular forces necessary to displace myelin by newly made layers of membrane from underneath might be too high. Moreover, several morphological features of CNS myelin suggest a different model, since the number of myelin layers can vary along the length of a single myelin sheath during its formation [119]. The “liquid croissant” model [120] proposes that after making axonal contact, the OL membrane spirally encircles the future internode and grows laterally, creating individual membrane layers. Experimental support for any of these models remained elusive until Snaidero and colleagues [121], using in vivo live-imaging, serial block-face imaging by focused ion beam, and high pressure freezing, showed that myelin growth occurs by the consecutive wrapping of the inner tongue (leading edge) around the axon and the coordinated lateral extension of the individual layers of myelin. Cytoplasmic sub-domains at the end of each myelin layer remain in close contact with the underlying axon moving laterally and around the axon towards the future node where they eventually form a set of closely apposed “paranodal loops” [121,122]. 

The search for factors that allow communication between glia and axons has focused on metabolites that are found to be altered in the course of demyelinating diseases. Endogenous metabolites might play an essential role in the NPC regulation of cellular identity and OPC differentiation. Differentially regulated metabolites from OPCs and mature OLs have been identified by mass spectrometry-based untargeted metabolomic and lipidomic analysis. A significant increase (up to 20-fold) in the levels of the amino sulfonic acid taurine was observed during the course of oligodendrocyte differentiation and maturation. The fact that addition of taurine to already selected promyelinating drugs (benztropine and miconazole) increased their efficacy supports its positive function in OPC differentiation. However, it has been noted that it is unlikely that taurine itself stimulates a pro-differentiation process by directly interacting with a receptor or alternative protein target but rather that exogenous taurine enhances OL differentiation by serving as a feedstock to produce a limiting metabolite pool [123]. N-acetylaspartate (NAA) is a molecule in the CNS that is used in clinics to assess neuro-axonal integrity and the integrity of oligodendrocytes in patients with demyelinating diseases [124]. N-acetylaspartate is synthesized by the enzyme N-acetyltransferase-8-like (NAT8L) in neurons and is catabolized by aspartoacylase (ASPA) in oligodendrocytes [125]. A hereditary genetic mutation of the gene coding for ASPA is known as Canavan Disease (CD), causing the loss of ASPA activity [126]. A number of studies support the conclusion that Canavan disease is a disorder of oligodendrocyte maturation that results in a loss of both oligodendrocytes and neurons [127] with consequent demyelination [124]. NAA at a concentration of 100 µM inhibits cAMP-mediated differentiation of glioma stem-like cells and oligodendrocyte progenitor cells, suggesting that it might have a role in maintaining an undifferentiated state through second messenger pathways [126]. A decrease in NAA is observed in MS patients, but this decrease is reversible in the acute stages of remission of the disease and therefore could be an indication of a dysfunction of neuronal metabolism prior to axonal degeneration [128]. How NAA might communicate with OLs remains to be determined. One possibility is that it could regulate lipid signaling required for myelination, but direct evidence is still missing. 

The Src-family of tyrosine kinases are also involved in neuron/OL signaling. The Src named Fyn is present in myelin-forming cells and is activated through stimulation of cell surface receptors in the CNS [129]. Fyn kinase activation in OLs promotes MBP mRNA translation in cytosolic ribonuclear particles where MBP mRNA is stored prior to axon engagement. Thus, Fyn kinase-mediated signaling appears to be one of the cues involved in the response to changes in the 3D environment where OLs mature [129,130]. Interestingly, this signaling responds to laminin, a component of the extracellular matrix (ECM), which could connect extrinsic and intrinsic cues through surface receptors and Fyn kinase activation. However, this kind of signaling must be restricted to brain axons, since it is not instructive for myelination in the spinal cord. In fact, spinal cord myelination is normal in Fyn KO mice, while myelin in the forebrain is diminished in mice lacking laminin α2 [1,131,132]. Synaptic-like communication between neurons and oligodendrocytes has been suggested to occur at distinct stages of OPC differentiation to regulate myelin plasticity, OPC number, choice of axons for ensheathment, myelin coverage of axons, and metabolic transfer [133,134]. Consistent with the possibility that nascent myelin sheaths might respond to axon inputs, the postsynaptic density protein 95 (PSD95) has been localized to myelin sheaths in zebrafish. In neurons, PSD95 anchors receptors, ion channels, and various synaptic signaling molecules at the postsynaptic membrane via its PDZ domains. Genetic manipulation of PDZ motifs in PSD95 indicates that its main function might be adhesion at the axon–myelin interface [135]. Another group [136] used EM to assess axon–OPC contacts in vitro. They showed that oligodendrocytes preferentially myelinate electrically active axons, but synapses from axons onto myelin-forming oligodendroglial cells are not required Although they observed presynaptic vesicles docked at axon–OPC contacts, postsynaptic density PSD95 protein was not identified and therefore they suggested that OL/axon junctions are not synaptic in structure. The discordance between these observations, according to Hughes and colleagues, might be due to the lack of EM-resolvable PSD until maturity, or that maturity of axon-OPC junctions was not achieved in culture in the samples analyzed by [136]. If dendrites and oligodendrocytes use synaptic mechanisms to establish stable contacts with axons, it is possible to envisage that psychiatric diseases based on genetic defects in the synaptic machinery (as observed in postmortem samples of schizophrenic and autistic patients’ white matter) might contribute to disease progression via reduced neuronal support or by altering the conduction velocity or disrupting oligodendrocyte maturation, myelination, or survival [135]. If the presence of synaptic-type junctions between axons and OLs will be confirmed, these proteins could represent important novel targets to contrast demyelination in these pathologies. 

### 3.1. In Vitro Mimicking of OPC Development in a 3D Environment

#### 3.1.1. Synthetic Axons

The idea to use reductionist approaches to study how OLs engage axons in vitro started in the early 1970s by attempting to mimic axons using glass fibers [137]. In the early 1980s, carbon microfibers were used to show that they can be wrapped by OL processes within four weeks from culturing [138,139]. In these tests, the synthesis of myelin-like membranes was further assessed by measurement of [^35^S] sulphate incorporation into sulfolipids: carbon microfibers could aid OPC aggregation and stimulate OPC differentiation in mixed neurons and glia primary cultures [140]. However, direct glass microfiber addition to pure OL cultures had instead little success due to the buoyancy of the microfibers that prevented contact with the cells [141]. Therefore, it became clear that it was necessary to design an apparatus to overcome these technical problems. One of the first systems developed was a culture chamber constructed to allow the close apposition of glass microfibers (coated with a glial cell matrix) and primary cultures of rat oligodendrocytes in a three-dimensional arrangement known as oligodendrocyte-fiber sandwiches. This system enabled the visualization of clusters of cells in contact with fibers by scanning electron microscopy (SEM), while images obtained through transmission electron microscopy (TEM) showed that they were not simply growing on the top of the fibers, but that the cells surrounded the fibers. OPC cells cultured in the presence of matrix-coated glass microfibers showed an increased production of sulfolipids that was at least partially dependent on the presence of the matrix coating [141]. 

These early works based on fragmented glass microfibers with a diameter of only 0.4 µm showed that OLs loosely wrap fibers of this size producing a non-compacted myelin. Since OLs tend to myelinate axons with larger diameters in vivo, in the following studies fibers were produced with a larger diameter using carbon (diameter 6.7 µm) to study electrical conduction [140], amorphous bundles (4 µm) diameter glass fibers, and a woven mesh prepared from 12 µm diameter polyglactin 910 (Vicryl) fibers [142]. Unfortunately, these approaches to create artificial axons were abandoned until Lee and colleagues [26] used electron-spinning technology to generate polystyrene (PS) microfibers with different diameters (0.2 to 4.0 µm) to definitively establish if OLs might have a size preference during myelination, and they demonstrated that axon engaging efficiency by OLs depends only on the axon diameter [26]. Polystyrene (PS), a thermoplastic aromatic polymer with a linear structure, is used in many scaffold production techniques (casting, electrospinning, and 3D printing) and remains the most used material that has now largely replaced glass for cell-based work [143,144,145]. 

Using PS fibers, it has been shown that rat primary oligodendrocytes indeed require a minimum fiber diameter threshold of 0.4 µm to initiate wrapping with an optimum diameter range of 2–4 µm to observe myelin-like segment formation. However, a correlation between the number of wraps and the caliber of the fiber was not observed, underlining the importance of the axonal signals for the maturation and compaction of myelin [26]. The physical mechanism mediating ensheathment leading to multiple membrane wrapping and compaction remains to be understood [99,100]. 

These studies showed for the first time that it is possible to decouple the two stages of myelination, engaging and “compaction”. Importantly, they showed that the initial stage of axon engagement does not require an active axon. Last but not least, they showed that it could be possible to select drugs promoting OPC differentiation up to axon engagement in a neuronal, microglia- and astrocyte-free environment. These findings gave an enormous impulse to the drug discovery field. To better mimic axon flexibility, Poly-L-Lactic acid (PLLA) has been used, as it facilitates cell anchorage in vitro. Cortical OPCs grown on PLLA microfibers differentiated into OLs and engaged microfibers producing multi-layered myelin sheaths [146,147,148,149]. Based on the non-neuron-instructed myelin formation hypothesis, 3D scaffolds formed by PLLA microfibers were used by Bechler and colleagues [1] as a neuron-free culture system to investigate the issue of how the morphology of the myelin sheath differs depending on the regional identity in the brain. They compared peripheral nervous system (PNS)-derived glial cells to oligodendrocyte-driven myelination. The same results were not obtained using Schwann cells, however. Although both OPCs and Schwann cells myelinated dorsal root ganglia (DRG) neurons, electron microscopy showed that the morphology of the resulting compacted membrane structures was different, revealing that OLs, in the absence of neuronal feedback, have regional identity and generate different sheath lengths that mirror their behavior in vivo. Furthermore, they investigated how axonal molecule laminin, known to enhance myelination through activation of the Src family kinase Fyn [129], modulates the OL-intrinsic myelination program. Cortical oligodendrocyte sheath lengths were unaffected by the addition of laminin. However, laminin coating increased the number of sheaths, which was not seen in the presence of the Fyn inhibitor PP2. Therefore, there is a difference between lengthening and thickening, which could be, respectively, neuronal-independent and dependent mechanisms during myelination [1]. 

Nerve stiffness modulates proliferation and differentiation of glial cells [27,150,151] and nerves are among the softest tissues in the body, with a Young’s elastic modulus E 0.1–1 kPa [152]. Using atomic force microscopy indentation measurements of demyelination tissues at the cellular scale in human and mouse demyelinated brains, Urbanski and colleagues [2] showed that acute and chronic demyelinated CNS lesions exhibit opposite elastic properties. OLs are mechano-sensitive cells and increasing the extracellular matrix (ECM) stiffness inhibits their differentiation. To better match the physical requirement of nerves [27], 3D printing technology is currently used to better control fiber alignment, diameter, and spacing features and to mimic the physiological axon orientation, allowing easier image acquisition and quantification of myelin ensheathment. 

Substrates with different orders of magnitude of stiffness have been tested. These were polydimethylsiloxane (PDMS) ink that forms elastic fibers (E = 976 ± 11 kPa), pHEMA-low-E and pHEMA-high-E, two inks that form viscoelastic hydrogels after hydration with low and high stiffness properties (E = 88 ± 10 and E = 333 ± 30, respectively), and two poly (HDDA-co-starPEG) resins of distinguishable stiffness (high E = 140 ± 35 kPa and very low E = 0.42 ± 0.14 kPa) [27]. This approach produced artificial axons with stiffness up to six orders of magnitude more compliant than state-of-the-art materials currently used for myelination assays. Thus, the ability of OLs to remyelinate axons might be affected also by the mechanical property of axons consequent to deposition of ECM, since ECM deposition changes over the time course of disease [2,27,31]. 

The main findings of these studies can be reassumed in the observation that the first stages of myelination are uncoupled from neuronal feedback and rely on the diameter and flexibility of axon fibers. 

#### 3.1.2. Extracellular Matrix Function in Axon Recognition and NPC Differentiation

The future of synthetic axon fibers probably depends on the ability to reconstitute the signals that are recognized by OLs during engagement. Chronic and acute demyelination of axons is associated with the deposition of different types of ECM. Patients exhibiting demyelination often show increased deposition of the ECM and chondroitin sulphate proteoglycans (CSP) at MS lesions [2,153,154,155]. Inhibitors of CSP deposition ameliorate remyelination [104]. It is known that the nature of the ECM strongly influences cell shape [156,157]. Laminin, a component of the ECM involved in OPC differentiation [158] was used for small fiber (0.2–0.4 μm) coating without increasing the OL myelin sheath [159]. Like laminin, neither nectin-like protein 1 (Necl1) [160] nor poly-lysine [26] affected myelination. Why coating of small fibers seems to be inefficient remains to be clarified. The use of gelatin to coat polycaprolactone (PCL) electrospun fibers increases the capacity to promote OL engagement with respect to naked PCL fibers [161]. 

Matrigel is a matrix that is found in liquid form at 4 °C, while at room temperature it solidifies and becomes a sort of gelatin [162,163] that has been well characterized by in-depth proteomic analysis [164]. The molecular components and the physical nature of Matrigel are important for its biological effects on cells. In addition to the main components of this matrix, laminin-111, collagen IV, entactin, and heparin sulphate [164], FGF, EGF, TGF beta, IGF, and PDGF growth factors are present and can vary from matrix to matrix, allowing a choice of the type of Matrigel most suitable for the growth of different cell types [165,166]. Matrigel provides the best imitation of in vivo cell environments for 2D and 3D culture applications. Despite its excellent biocompatibility and cytocompatibility, its derivation from murine sarcoma cell lines makes Matrigel unfit for clinical applications. It would be interesting to realize a Matrigel coating functionalized to allow the growth of oligodendrocytes, since applications of this type have not yet been studied sufficiently.

Hydrogel and Matrigel have been used to support NPC growth to improve the therapeutic efficacy of transplants and improve the outcome of focal cerebral ischemia in rodents [167]. Human NPC implants, derived from embryonic stem cells (ESCs) grown in Matrigel, led to a reduction in the size of the brain cavity caused by ictus, increased the survival of transplanted cells, the acquisition of neuronal properties, and thus improved neural differentiation [168]. Hydrogels have numerous characteristics that make them excellent biocompatible materials for neurological cultures. Due to their hydrophilic nature, they have a high-water content (>90%) that promotes cell culture and also offer a range of mechanical characteristics for which they can be designed to mimic many organic tissues [169]. The most important synthetic hydrogel is polyethylene glycol (PEG) which offers a wide variety of configurations [154]. For example, a fully synthetic PEG-based Anisogel (sPEG) has been developed and adapted with recombinant fibronectin fragments (FNIII9 *-10/12-14) to induce the aligned growth of nerve cells [170]. The sPEG hydrogel cross-links enzymatically with activated factor XIII, similar to fibrin, showing for the first time that a fully synthetic injectable Anisogel is capable of inducing nerve growth in a way that is oriented parallel to the elements. This concept represented an important step towards the clinical translation of a regenerative treatment of hierarchically structured tissue [170]. Additionally, the 3D Hyaluronic acid (HA) hydrogels are a useful platform for studying OPC behavior, and their study has shown that OPC growth/metabolic health may be favored in lower stiffness microenvironments mimicking brain tissue mechanics [171]. HA hydrogels engineered to match brain tissue stiffness also support NPC viability and neuronal differentiation [172].

A further level of potential development of fiber coating could be the use of graphene. Graphene oxide (GO) has been used to improve differentiation of OPCs into mature OLs. The functional groups of GO interact better than graphene with lipid membranes and other molecules, especially when exploited as a drug carrier [116,173,174]. This feature makes it interesting for fiber-OL drug tests, because the drug could be directly embedded in the fiber mesh. So far, graphene has been used for biotechnological applications and stimulation of oligodendrocyte growth. The large 2D aromatic surface of graphene makes it an ideal substrate for the adsorption of certain biomolecules, and ssDNA can be strongly interfaced on the graphene surface [175,176]. A graphene-based engineered scaffold able to induce differentiation of NSCs into OLs is the conducting polymer (3,4-ethylenedioxythiophene) (PEDOT) and graphene oxide (GO) composed nanosheets (GO/PEDOT). PEDOT is a conductive polymer, which has received a lot of attention for its high electrical conductivity and its chemical stability. Because the subpopulation of OLs from NSCs on GO/PEDOT nanofibers is very low, Weaver and Cui [177] modified GO/PEDOT with the OL mitogen PDGF-CL to improve the levels of the oligodendrocyte cell subpopulation. This strategy, furthermore, significantly increased the extent of OL process outgrowth compared to GO/PEDOT nanofibers [177,178]. The use of nanofibers coated with GO, such as PCL-GO nanofibers or GO/PEDOT nanofibers, also induced morphological changes and protein expression indicative of the differentiation into mature OLs. This material, thanks to its multiple and interesting properties, ease of processing, 3D printability, and electrical conductivity, seems to be quite promising for 3D fiber-support production. Moreover, its ability to act as a drug carrier should not be neglected in view of future therapies for drug discovery analyses. In summary, the development of artificial axons alone or coated with ECM-like materials could facilitate the understanding of some key aspects of OPC differentiation and OL-mediated myelination processes. Several ECM molecules have been used as a substrate for OPC cultures [179,180], but little information is available about their effects on OPC growth. OL-mediated axon engagement can be divided in two phases, an axon-dependent and axon-independent phase. The axon independent phase recognizes the diameter and flexibility of the axon fibers. The second phase recognizing ECM material and conductivity is also likely to play a role in communication. Soluble cofactors produced by neurons might contribute to this communication, and the use of synthetic axons will further contribute to our understanding of the biological basis required for axon myelination. Last but not least, studies performed using synthetic axons have led to a proliferation of drug discovery projects in academic and non-academic environments that made use of chambers containing fibers [37,98] or multiwall plate with micropillar cones [29,30], providing the basis for the first large drug discovery programs in academic environments, avoiding animal use at early stages of the drug discovery pipeline. 

## 4. Identification of Promyelinating Drugs Using Phenotypical Screen

### 4.1. Cellular Models to Study Remyelination and Suitable for Phenotypical Drug Library Screens 

The search for remyelinating drugs requires appropriate cellular models. In the case of CNS remyelination, the main problem, from the drug discovery point of view, was the identification of oligodendrocyte cell lines that could reasonably recapitulate one or more aspects of OPC maturation during drug testing. Cell biology and neurobiology know-how was therefore directed towards creating a cellular model that could recapitulate the fundamental aspects of OPC differentiation in a time compatible with large drug screens. Essentially four main in vitro models (Table 2) were used for phenotypical screens: (1) zebrafish [181], (2) primary OPCs derived from rat optical nerve [6,29,30,34]; (3) mouse embryonic oligodendrocyte cell lines (Oli-neu, Oli-neuM) [33,37], and (4) OPC-derived mouse epiblast stem cells [7]. 

The zebrafish *Danio rerio* larva was used as a developmental model of myelination due to its excellent imaging capabilities combined with easy genetic manipulation [182]. The major biochemical difference between zebrafish and mammalian myelin is the presence of protein zero (P0) as a major CNS myelin protein in zebrafish, rather than PLP in mammals. The zebrafish P0 gene shows greater sequence conservation and promoter region match with the mammalian PLP gene rather than the mammalian P0 gene [183,184], and there is not a clear biochemical distinction between oligodendrocytes and Schwann cells in zebrafish [184]. 

The identification of the NPC niche reactivated during remyelination in the adult brain [77,185] resulted in the possibility of purifying the specific OPC lineage to be used in phenotypical drug screens. This finding, however, led to few drug discoveries programs due to the complexity of the OPC purification technology and culturing for large drug screens [6,29]. 

The third approach used mouse immortalized embryonic cell line (Oli-neu and Oli-neuM). The Oli-neu cell line (Cellosaurus CVCL_IZ82 [186]) is a well-characterized oligodendrocyte cell line used to study myelin gene expression [187], but it does not differentiate up to the stage of axon engagement [188]. Another limitation of the Oli-neu cell line in drug screening is its pro-apoptotic behavior as poorly expressed in the MyrF gene. Following the identification of the key transcriptional factor MyrF, which marks the boundary between OPCs and pre-myelinating OLs [96], the Oli-neuM cell line stably expressing MyRF was constructed (Cellosaurus CVCL_VL76). Oli-neuM can be easily and reproducibly cultured in multi-well plates, and it has been used in drug phenotypical screens [33], validation tests, and artificial axon engagement studies. Moreover, RNA extraction, gene silencing, and other molecular manipulations required for drug activity testing in vitro have been developed for this cell line [37]. 

The fourth approach used relied on the development of a methodology to derive OPCs from mouse epiblast stem cells [7]. Epiblast is a tissue of the post-implantation embryo that generates the embryo. Given appropriate encouragement, it can differentiate into virtually any cell type. Human embryonic stem (ES) cells are potentially important in therapy, because they are pluripotent and capable of differentiating. However, it was not possible to develop suitable screening technologies until it was shown that mouse epiblast stem cells pluripotency (EpiSC) [189] was based on morphological, molecular, and functional criteria and shares properties with human hESCs [190]. The technology for culturing OPCs derived from EpiSCs [191] has overcome the inefficiency and difficulty of culturing primary OPCs in vitro and led to the development of several platforms for drug screening [7,98]. 

After the development of the cellular models and platforms for OPC growth and differentiation, several groups used them for drug repurposing campaigns to identify promyelinating drugs. Despite the different cellular models used, all selected the similar classes of compound: glucocorticoids (clobetasol, halcinonide) [7,33], EGFR-TKIs (erlotinib, gefitinib, imatinib) [33,192], benztropine [6], and antifungal agents (miconazole and clotrimazole) [7,33] (Table 1 and Table 2). The success of these studies showed that successful drug discovery for remyelination rely on a combination of the identification of the appropriate OPC cellular model and the development of technology for their isolation and culturing for large scale drug discovery programs.

**Table 2:** Models for drug screening: advantages and disadvantages and selected drugs.

Model for Drugs Screening	Advantages	Disadvantages	Drugs Identified in Phenotypical Screen	Ref.
**Zebrafish Danio rerio larva**	Excellent visualization of myelination; easy genetic manipulation	Biochemical and genetic difference among species	Src kinase inhibitor PP2; a biogenic ammine and a Thioxanthene, Fenofibrate, Gemfibrozil.	[181,182,183,184,193]
**Primary Cells Culture**	Genetic and biological fidelity	Complex isolation; difficult to scale up for drug screening	*Regulators of muscarinic acetylcholine signaling*: Benztropine, Clemastine, Donepezil, Oxybutynin, Vesamicol, Ipratropium. *Estrogen receptor modulators (SERMs)*: Raloxifene, Toremifene, and Tamoxifen. *Tricyclic antidepressant molecules*: Perphenazine, Prochlorperazine, Fluphenazine, Trifluorperazine, Quetiapine fumarate. *Non-tricyclic antidepressants*: Perospirone, Escitalopram, Citalopram, Metylperon, and Bupropion. *Regulators of adrenergic receptor pathway*: Salmeterol, Betaxolol, Esmolol. *Ion channel modulators*: Ifenprodil, Benproperine, Proxymetacaine, Dofetilide, Dimethylphenylpiperazinium. *Antifungal agent:* Bifonazole.	[6,29,30,34]
**Oli-neu,** **immortalized oligodendrocyte cell line**	Well characterized at molecular level for Myelin protein trafficking and lipid signaling	Difficult to culture; Pro-apoptotic behaviour. Cannot be used in axon engagement tests. Mouse cell line	*PKA pathway agonist*: dbcAMP, Forskolin. *Nuclear receptor ligand*s: Nr3C1: Dexamethasone, Hydrocortisone, Budesonide, 1,3-cis-Retinoic acid: RxRs. *ErbB inhibitor:* PD174265, 4557W. *Nucleoside analogs*: Ribavirin, Lefluonomide.	[186,187,188,192]
**Oli-neuM, immortalized oligodendrocyte cell line**	Easy to culture in drug screening; stably expressing MyrF gene. It differentiates till axon engagement. Suitable for in vitro synthetic axon engagement tests	Mouse cell line	*Glucocorticoids acting as Smo agonists:* Clobetasol, Halcinonide. *EGFR inhibitors*: Gefitinib Erlotinib. *Antifungal agents:* Clotrimazole, Itraconazole. *Immunosuppressant*: Azatriopina.	[33,37,186,187,188]
**OPCs from mouse** **epiblast** **stem cells**	Easy manipulation; Fast results; high reproducibility; easy to scale up for drug screening	Mouse cell line	*Glucocorticoids*: Clobetasol, Bethamethasone, Methilprendisolone. *Antifungal agents:* Clotrimazole, Miconazole, Bifonazole, Ketoconazole.	[7]
**Human iPSC-derived OPC (HiPSC)**	Genetic and biological fidelity; iPSC from patient; they can recapitulate disease defects	Derivation of OPC from iPSC requires good technical skill; it requires time. Large drug screens not performed as yet	*Immunomodulatory treatment* of PBMC	[4,194,195,196]
**Organoids**	Promising close model to resemble the development, composition, architecture, and partially the function ex vivo of the original human tissue	Require long differentiation time, technology for culturing; expensive. Do not recapitulate brain structures		[197,198]

### 4.2. Drug Repurposing Strategies in Remyelination 

The technique of repurposing drug libraries of biological active compounds allows the identification of new therapeutic targets for drugs already in clinical use and thus reduces the toxicological and pre-clinical tests necessary for the validation of a therapy. Drug repurposing has therefore become a productive approach for drug discovery in academic environments, because it provides a novel way to explore old drugs for new use and a relatively small library to be screened [199,200]. Generally speaking, traditional drug repositioning studies focus on uncovering drug effects based on their mode of action or the knowledge of the defective disease process [201]. Preliminary knowledge on the drug safety and effectiveness in biological sample reduces drug development costs by lowering the risk of drug failure at later stages of drug development. Moreover, phenotypical screens are based on the identification of drugs that restore lost function. Thus, if the appropriate disease relevant model is used, phenotypical screening allows the selection of compound with high probability to function in animal models. This approach is certainly quicker and cheaper than the traditional target-based screening approaches, since molecules with similar pharmacological profiles might not act equally in a cellular contest. On the other hand, by targeting the “defective process” and not only one “target” in the cell, the search for the effective target at the effective dosage in the cell might be the bottle neck of the drug discovery campaign [200]. Moreover, the drug phenotypical screening strategy is unlikely to define the dosage required in vivo, as many other factors might affect the ability of selected drugs to arrive to the compartment of action in vivo [202]. A significant challenge in drug repositioning is to distinguish between primary and secondary molecular targets and identify molecular action; therefore, several strategies are often employed, such as reducing the pharmacological complexity of the drug library using computational pre-screening methods, using several rounds of secondary validation tests prior to selecting lead candidates, or using metabolomic and transcriptomic analyses to better understand the mechanisms of how the drug acts in relevant models [200,203].

Remyelination phenotypical screens and drug repurposing have certainly made a difference in increasing the panel of drugs to bring to clinical attention. It is too early to say if these campaigns have also increased success at the clinical level, since only clemastine and quetiapine fumarate (29) have reached clinical attention (Table 1). The list of compounds identified is quite long (Table 2). 

Some compounds were selected irrespective of the OPC cell based assays or FDA library used for the phenotypical screen, suggesting that they are among the most effective in remyelination, possibly by targeting conserved mechanism of OPC differentiation. Among them are RxRgamma agonists (bexarotene, 1,3-cis retinoic acid), glucocorticoids receptor (GR) agonists (dexamethasone, clobetasol, halcinonide budesonide), glucocorticoids with GR and Smo agonist activity (clobetasol, halcinonide), EGFR/ErbB inhibitors (gefitinib, erlotinib, PD174265, 4557W), and antifungal agents (clotrimazole, itraconazole, miconazole, bifonazole). In an attempt to clarify their mechanism of action, in a cumulative study, the antifungal agent (miconazole, clotrimazole, budesonide), the antihistaminic clemastine, and the selective M1 muscarinic acetylcholine receptor antagonist with antihistamine activity, benztropine, were studied in parallel. They were all found to cause 8,9 unsaturated sterols accumulation, in addition to acting on their known targets [98]. Thus, it appears that each drug identified in phenotypical screen targets multiple pathways. This observation suggests that the most effective drugs promoting remyelination in vivo might modulate more than one pathway/target, leading to OPC differentiation. In this respect, compounds identified using target-based pharmacology and those identified using phenotypical-based screens might lead to different efficacy results at clinical level. So far, both methods have led to active compounds acting in animal model for demyelination. However, their efficacy in clinic remains to be determined. 

A second, but not less important, result of the drug repurposing campaign has been that we can use these drugs as a toolbox to clarify the mechanism of how OPCs mature and engage axons in a controlled and well-defined environment [4,37,98]. These studies will certainly produce novel drug targets for future screening programs.

### 4.3. Human iPSC-Derived OPCs and Organoids

Neural specification of human pluripotent stem cells (hPSCs) represent the opportunities for studying human neural development using patient tissues. Induced pluripotent stem cells (iPSCs) have emerged as promising cell-based assays to be used in drug screenings to overcome the obstacle of isolating OPCs from human samples and better reproduce disease relevant features, as they can be obtained from somatic cells of patients. The recent development of human iPSC-derived OPCs has further improved the cellular tool-kit available for drug discovery and in vitro engagement studies [4,197,204]. Human induced pluripotent stem cell-derived oligodendrocytes (HiOL) have been recently obtained from RRMS patients and healthy individuals, providing an answer to the question of whether MS-derived OPCs have an intrinsic genetic problem of differentiation [4]. In this study, the intrinsic or extrinsic factors contributing to impaired oligodendrogenesis in RRMS vs. healthy individuals were examined. The global comparison of the respective proteomes revealed a high level of similarity between healthy and RRMS HiOLs. A significant oligodendroglia differentiation block was observed only after application of supernatants of activated peripheral blood mononuclear cells to healthy and RRMS HiOLs. These observations supported the view that the major obstacle to OPC differentiation derives from the inflammatory environment in which MS OPCs develop compared to healthy individuals. Surprisingly, a number of drugs previously shown to be active in remyelination in the EAE animal model, such as benztropine [6], miconazole, and clemastine [7], failed to overcome the HiOPC block, supporting the view that the inflammatory environment but not an intrinsic block of OPC maturation differentiates RRMS HiOL development from that of healthy individuals [4]. However, this study cannot respond to the question if OPC heterogeneity among MS individuals plays a role in disease progression. Moreover, many of the selected drugs in phenotypical screens belong to the class of anti-inflammatory drugs (Table 2). Unfortunately, these drugs were not selected in this study. Future studies using HiOPCs obtained from different classes of MS patients and a test on a larger number of drugs, possibly comprising anti-inflammatory compounds, might better clarify these issues. 

The evolution of stem cell manipulation techniques such as the derivation of HiPSCs has led to the development of new human disease models and the development of more predictive pharmacological screening platforms [191,192]. Windrem and colleagues [205] created human glial chimeric mice using iPSCs derived from juvenile-onset schizophrenia (SCZ) patients. These chimeric mice showed aberrant migration of SCZ-derived iPSCs and reduced and insufficient white matter expansion compared to the control mice with a consequent reduction of the differentiation into oligodendrocytes, leading to hypomyelination and abnormal behavioral phenotypes. The cellular model might open the way to studying the remyelination aspects in schizophrenia [205]. A further promising development of HiPSC culturing technology has been recently applied to the construction of printed supports made of microsphere-laden bioink used to bioprint HiPSC-derived NPCs and to deliver drugs [32]. This work is based on evidence that culturing HiPSCs with small molecule morphogens can shorten significantly their differentiation in the cell type of interest, as this can take months. Recently, neuronal stem cells derived from HiPSCs were bioprinted with relatively high levels of viability into structures that resemble the spinal cord [206]. In a further development, this 3D bioprinting technology can be envisaged to be used for promyelinating drug release by microspheres to HiOPCs obtained from AD patients, providing a tool for screening drugs in a disease relevant model [32].

Human-derived organoids also offer several promises for the further development of remyelination models for drug discovery [207]. Their therapeutic promise is of great interest for the pharmaceutical industry, although it is too early to determine what impact they will have in improving the clinical applicability of identified compound or to validate those already suggested for remyelination therapy [207,208,209]. Stem cells inherently have the ability to self-assemble into complex structures to summarize the structural characteristics and cell–cell interactions of tissues thanks to growth factor morphogen addiction in a tissue-specific manner [208]. The main advantage of organoids is the fact that they could represent a three-dimensional model, potentially derived from patient iPSC, that might more closely resemble the development, composition, architecture, and partially the function ex vivo of brain human tissue [197,198]. Last but not least, organoids could offer a source for cell therapies, a potential alternative for whole-organ transplantation, and in combination with novel genome-editing techniques, open up the possibility of performing gene correction and clone selection prior to autologous transplantation [210,211]. OLIG2-GFP knockin human pluripotent stem cell (hPSC) reporter lines were used to generate brain region-specific dorsal forebrain organoids and ventral forebrain organoids by inhibiting or activating SHH signaling pathway [212]. Furthermore, organoids have been used to better understand the molecular and genetic mechanisms of autism and Alzheimer’s disease [190,213]. Cortical organoids derived from HiPSCs of AD patients with presenilin-1 mutation have been investigated to clarify the AD-related inflammatory responses, matrix remodeling, and the responses to DAPT, heparin, and heparinase treatments [213].

## 5. Conclusions

During the past ten years the collective efforts of neurobiologists, material engineers, and pharmacologists have provided the requirements to support large drug discovery programs for remyelination agents. The substantial differences in adult remyelination processes between man and rodent represent an important obstacle for recreating the complexity of the human brain in vitro. The observed oligodendrocyte heterogeneity at the lesion and the changes in axon stiffness and viscosity during pathology progression support the view that OPCs as well as OLs sense their 3D environment. Changes in any of these factors can contribute to regulating the ability OPCs and OLs to remyelinate axons and, possibly, the efficacy of remyelination agents during disease progression or aging. Therefore, further efforts are needed to understand the mechanism that promotes OPC recruitment and OL differentiation at the lesion. The creation of novel 3D models combined with the use of patients iPSCs and organoids should provide new avenues for the discovery of remyelination agents. 

## Figures and Tables

**Table 1:** **Drugs promoting CNS remyelination in preclinical studies tested in clinical trials.** List of drugs implicated in remyelination currently in clinical trials. The compounds are listed on the basis of the clinical study phase. Data were obtained from https://clinicaltrials.gov/ct2/home (accessed on 10 November 2020) and https://www.clinicaltrialsregister.eu/ctr-search/trial/2014-003145-99/GB (accessed on 10 November 2020) databases.

Drug Name	Drug Class	Target/Evidence of Action in Remyelination	Drug Identification Method	Clinical Trial Phase	Ref.
**IRX4204**	Second generation RXR agonist	It agonizes RXR-γ, a positive regulator of the differentiation and remyelination of endogenous precursor cells of oligodendrocytes	Target-based/In vivo studies	Phase 1 ClinicalTrials.gov Identifier: NCT02438215	[38,39]
**T3**	Thyroid Hormone	T3 is required for central CNS myelination during development, and CNS remyelination in animal models of MS	Micropillar-based, drug repurposing phenotypical screen	Phase 1 ClinicalTrials.gov Identifier: NCT02760056	[29,40,41,42]
**Quetiapine fumarate**	Dibenzothiazepine	Atypical antipsychotic drug Used in the symptomatic treatment of schizophrenia and bipolar disorders. It has a role as a serotonergic antagonist, a dopaminergic antagonist, a histamine antagonist, an adrenergic antagonist, and a second generation antipsychotic. It stimulates proliferation of OPC and their differentiation oligodendrocytes. It increases SOD1 activity and the scavenging of free radicals, alleviating oxidative stress.	Micropillar-based, Drug repurposing phenotypical screen	Phase 1/2 ClinicalTrials.gov Identifier: NCT02087631	[29,43,44,45,46]
**Metformin**	Biguanide hypoglycemic agent used in the treatment of non-insulin-dependent diabetes mellitus	Metformin is associated with a reduction in MS disease activity, but the potential mechanisms underlying this anti-inflammatory effect have not yet been clarified.	Not specified/in vivo validation	Phase 1/2 ClinicalTrials.gov Identifier: NCT04121468	[47]
**GSK239512**	Small molecule	Histamine H3 antagonist, orally available, assessed for modulating cholinergic and monoaminergic neurotransmission in AD. In RRMS, it has been tested for enhancing remyelination.	Not specified	Phase 2 ClinicalTrials.gov Identifier: NCT01772199	[48]
**Opicinumab**	Monoclonal antibody	LINGO-1 antagonist. SYNERGY study did not show a significant dose-linear improvement in disability compared with placebo in patients with RRMS.	Target-based. LINGO-1 is a cell-surface glycoprotein selectively expressed on CNS neurons and OL. It inhibits oligodendrocyte differentiation, myelination.	Phase 2 ClinicalTrials.gov Identifier: NCT01864148 Phase 2 ClinicalTrials.gov Identifier: NCT03222973	[49,50]
**Clemastine**	Anti-histamine.	Competitively bind to histamine receptor H1 sites. It promotes oligodendrocytes differentiation and myelination by inhibitor of cytochromeP450. It may interfere with other drugs metabolized by this isozyme.	Micropillar-based, drug repurposing phenotypical screen	Phase 2 ClinicalTrials.gov Identifier: NCT02040298	[29,51]
**Bexarotene**	Synthetic retinoid- RXR agonist	It agonizes RXR-γ, a positive regulator of the differentiation and remyelination of endogenous precursor cells of oligodendrocytes	In vitro/in vivo Studies	Phase 2 EudraCT Number: 2014-003145-99 *	[38,52,53]
**Domperidone**	Peripheral Dopamine receptor antagonist	It increases prolactin levels and this may improve remyelination	Not specified, validated in vivo	Phase 2 ClinicalTrials.gov Identifier: NCT02493049 (RRMS) Phase 2 ClinicalTrials.gov Identifier: NCT02308137 (SPMS)	[54,55]
**Pioglitazone**	PPARƴ agonist	Anti-inflammatory effects in glial cells. Delays onset and reduces severity of clinical symptoms in EAE mice	In vitro/in vivo studies	Phase 2 ClinicalTrials.gov Identifier: NCT03109288	[56,57]
BIIB061 **	Small molecule produced by Biogen	Undisclosed	Not specified	Phase 2 ClinicalTrials.gov Identifier: NCT04079088 **	[58,59]
**Simvastatin**	Statin	Inhibits 3-hydroxy-3-methylglutaryl co-enzyme A reductase, restricting synthesis of cholesterol and the post-translational lipid attachments, isoprenoids. Reduces initial disease severity in an EAE animal mode following short-term statin therapy. 24-month MS-STAT phase 2 trial. High dose simvastatin significantly reduced the annualized rate of whole brain atrophy in patients with secondary progressive multiple sclerosis (SPMS).	Synthetic derivative of a fermentation product of *Aspergillus* *terreus*	Phase 2 ClinicalTrials.gov Identifier: NCT00647348 Phase 3 ClinicalTrials.gov Identifier: NCT03387670	[60,61]

* A randomized placebo-controlled study of the safety and tolerability of a retinoid-X receptor agonist’s ability to promote remyelination in people with RRMS already on interferon-beta therapy. ** Study to Evaluate the Efficacy and Safety of Oral BIIB061 as Add-On Therapy to Interferon-Beta 1 therapies in RMS.

## Data Availability

Data in Table 1 were obtained from https://clinicaltrials.gov/ct2/home (accessed on 10 November 2020) or https://www.clinicaltrialsregister.eu/ctr-search/trial/2014-003145-99/GB (accessed on 10 November 2020); and from given references.
